# Functional analysis of a first hindlimb positioning enhancer via *Gdf11* expression

**DOI:** 10.3389/fcell.2024.1302141

**Published:** 2024-03-15

**Authors:** Seiji Saito, Utsugi Kanazawa, Ayana Tatsumi, Atsuo Iida, Tatsuya Takemoto, Takayuki Suzuki

**Affiliations:** ^1^ Department of Animal Sciences, Graduate School of Bioagricultural Sciences, Nagoya University, Furo-cho, Chikusa-ku, Nagoya, Japan; ^2^ Division of Biological Science, Graduate School of Science, Nagoya University, Nagoya, Japan; ^3^ Department of Biology, Graduate School of Science, Osaka Metropolitan University, Osaka, Japan; ^4^ Institute for Advanced Medical Sciences, Tokushima University, Tokushima, Japan

**Keywords:** *Gdf11*, enhancer, hindlimb, sacral vertebrae, morphogenesis

## Abstract

During the early development of tetrapods, including humans, the embryonic body elongates caudally once the anterior-posterior axis is established. During this process, region-specific vertebral morphogenesis occurs, with the determination of limb positioning along the anterior-posterior axis. We previously reported that *Gdf11* functions as an anatomical integration system that determines the positioning of hindlimbs and sacral vertebrae where *Gdf11* is expressed. However, the molecular mechanisms underlying induction of *Gdf11* expression remain unclear. In this study, we searched for non-coding regions near the *Gdf11* locus that were conserved across species to elucidate the regulatory mechanisms of *Gdf11* expression. We identified an enhancer of the *Gdf11* gene in intron 1 and named it highly conserved region (HCR). In HCR knockout mice, the expression level of endogenous *Gdf11* was decreased, and the position of the sacral-hindlimb unit was shifted posteriorly. We also searched for factors upstream of *Gdf11* based on the predicted transcription factor binding sites within the HCR. We found that inhibition of FGF signaling increased endogenous *Gdf11* expression, suggesting that FGF signaling negatively regulates *Gdf11* expression. However, FGF signaling does not regulate HCR activity. Our results suggest that there are species-specific *Gdf11* enhancers other than HCR and that FGF signaling regulates *Gdf11* expression independent of HCR.

## 1 Introduction

Tetrapods, including humans, possess vertebrae that extend from the cranial to the caudal position. These bones are categorized as cervical, thoracic, lumbar, sacral, and caudal. The number of vertebrae is characterized by a vertebral formula: for example, C7, T12, L5, S7, and Co4 in humans, and C7, T13, L5, and S4 in mice. Of the free limbs, the forelimb is located at the first thoracolumbar vertebra, and the hindlimb is directly articulated or fused to the sacral vertebrae via the pelvis. Thus, the vertebral formula, together with the positions of the limbs, is diverse in tetrapods.

During early development, the body elongates along the anterior-posterior axis, with cells proliferating on the caudal side. During this process, prospective vertebrae derived from the presomitic mesoderm (PSM) develop as somites. Following this, each somite acquires positional information by receiving various signaling inputs following the region-specific expression of Hox genes, which regulate region-specific vertebral morphogenesis along the anterior-posterior axis (Benazeraf and Pourquie, 2013; Neijts et al., 2014; Kimelman, 2016).

For continuous extension of the PSM posteriorly, axial stem cells located at the posterior end of the embryo must maintain an undifferentiated state to contribute to the PSM cell population. The FGF and WNT signaling pathways maintain the undifferentiated state of axial stem cells (Wilson et al., 2009; Gouti et al., 2014; Henrique et al., 2015). *Fgf4* and *Fgf8* are FGF signaling ligands expressed in the posterior region of the embryo (Naiche et al., 2011; Boulet and Capecchi, 2012). Mice deficient in both *Fgf4* and *Fgf8* or their receptor *Fgfr1* show not only defects in the posterior region of the body but also homeotic alterations in the vertebrae (Partanen et al., 1998; Boulet and Capecchi, 2012). These results indicate that FGF signaling is essential for posterior elongation of the body and is involved in determining vertebral identity along the anterior-posterior axis. WNT signaling is also essential for the formation of the posterior regions of the embryo and vertebral patterning. *Wnt3a*, as well as *Fgf8*, is expressed at the posterior end of the embryo and *Wnt3a*-deficient mice do not form structures behind their forelimbs, including the second cervical vertebra (Takada et al., 1994; Ikeya and Takada, 2001). Mice with mutated *Lrp6*, a co-receptor of *Wnt3a*, and mice with mutations in both *Tcf1/Lef1*, a transcription factor downstream of *WNT* signaling, also show phenotypes similar to those of Wnt3a mutant mice (Galceran et al., 1999; Pinson et al., 2000). These results suggest that the FGF and WNT signaling pathways are essential for the formation of the posterior regions of the embryo and normal morphogenesis of the vertebrae Saito and Suzuki, 2020.

We previously reported that expression onset of *Gdf11*, a secreted factor belonging to the TGF-β superfamily, during the early developmental stage determines the positions of the hindlimbs and sacral vertebrae (Matsubara et al., 2017). *Gdf11* expression was first observed in the posterior axial mesoderm (pAM), which is located posterior to the PSM. The GDF11 protein is secreted into the adjacent lateral plate mesoderm (LPM), which gives rise to the hindlimb bud. *Gdf11* induces Hox gene expression in both the PSM and LPM and regulates the anatomical integration system, which is responsible for the positioning of the hindlimb at the sacral vertebra, following coordinated formation of the sacral-hindlimb unit. The onset of *Gdf11* expression is unique to each species; for example, six pairs of somites form at somite stage (ss)6 in soft-shelled turtles, ss7 in mice, and ss10 in chickens. In *Gdf11*-knockout mice, the position of the sacral-hindlimb unit is posteriorly shifted by six to eight vertebrae due to the presence of additional thoracic and lumbar vertebrae (McPherron et al., 1999). We reported a positive correlation in tetrapod species between the number of somites formed when *Gdf11* expression first occurred and the number of somites from the head to the hindlimb buds. In other words, the more somites present from the head to the hind limb buds, the latter is the onset of *Gdf11* expression. Therefore, identification of the molecular mechanism underlying the induction of *Gdf11* expression is essential for understanding the precise position of the sacral hindlimb unit along the anterior–posterior axis during development.

Here, we report a *Gdf11* enhancer region conserved among tetrapods and show that it is essential for endogenous *Gdf11* expression and the determination of the position of the sacral-hindlimb unit. Furthermore, we found that FGF signaling regulated *Gdf11* expression.

## 2 Materials and methods

### 2.1 Embryos

Animal handling and all experimental procedures were conducted in accordance with the guidelines of the Osaka Metropolitan University Animal Care and Use Committee. Animal experiments were approved by the Animal Experiment Committee of the Graduate School of Sciences, Osaka Metropolitan University. The protocols for embryo research were approved by the Osaka Metropolitan University Animal Experiment Committee (approval number: S0086).

Mouse (*Mus musculus*) embryos were collected from mice crossed with the enhancer knockout mice and/or *Gdf11* ^*+/−*^mice. Chicken (*Gallus gallus*) fertilized chicken eggs were purchased from Yamagishi (Ogaki, Japan) and Takeuchi Chicken Farms (Nara, Japan). Embryos were staged as described by Hamburger and Hamilton (1951).

### 2.2 *in ovo* electroporation

Electroporation was performed according to previously published protocols (Sato et al., 2002; Nakaya et al., 2004), with some modifications. After incubating the eggs until HH 8, 5 mL albumin was removed through a hole created at the broader end of the egg. Subsequently, the eggshells were removed using curved scissors. The vitelline membrane was removed using a tungsten needle to expose the surface of the embryo. Then, 100–200 μL black ink/phosphate buffered saline (PBS) solution (1:40 ink in PBS; rOtring, Germany; S0194660) was injected beneath the embryo using a 1 mL syringe. The *tk-EGFP* vector containing candidate enhancer regions and/or the pCAGGS-*mCherry* vector was diluted with distilled water to a final concentration of 1 μg/μL and mixed with Brilliant Blue G (diluted in water to a final concentration of 0.1%; Tokyo Chemical Industry, Japan; B1146). pCAGGS-constitutively active (CA) *Mek1* or CA-*β catenin* was diluted with distilled water to a final concentration of 1 μg/μL.

An L-shaped platinum anode (BEX, Japan; LF613P3) was inserted into the hole to inject the black ink/PBS solution. The tip of the platinum anode was fixed slightly behind the Hensen’s node and at a depth of 2 mm from the embryo. The DNA solution was applied over the caudal lateral epiblast (CLE) posterior to the Hensen’s node at ss4 (HH 8) of the chick embryo (Sato et al., 2002) using a glass pipette. A sharpened tungsten needle (The Nilaco Corporation, Japan; W-461267) of the cathode was placed over the CLE within the prospective PSM region. Electric pulses (25 ms pulse-on and 50 ms pulse-off at 7 V, three times) were applied using a GEB14 electroporator (BEX). After electroporation, the cathode was gently withdrawn from the amnion, and 30 μL of a penicillin-streptomycin solution (1:100 penicillin–streptomycin in PBS; Invitrogen, Thermo Fisher Scientific, MA, USA; 15,140-122) was added near the embryo. The eggshell window was firmly sealed with plastic tape. The following regions were amplified by PCR using specific primer pairs and inserted upstream of *tk-EGFP*: HCR (F-5′- TGT​ACG​TCT​CAG​CAA​CTC​AGC​TGA​C -3′, R-5′- CAGGGGCAGGAGGTTGGG -3′), a region (F-5′- GAC​AGG​AGC​GGC​TTT​GAA​ATT​TTA​TGG​CCT​GGA​AAA​TCC​AGG​CC -3′, R-5′- GGC​CTG​GAT​TTT​CCA​GGC​CAT​AAA​ATT​TCA​AAG​CCG​CTC​CTG​TC -3′), b region (F-5′- GGT​TTT​ATG​GCT​CTG​AAC​AGA​AGG​GGG​GGC​TGG​TTT​ATT​GGC​AGA​TGG​GTC​ATA​AAA​AGC -3′, R-5′- GCT​TTT​TAT​GAC​CCA​TCT​GCC​AAT​AAA​CCA​GCC​CCC​CCT​TCT​GTT​CAG​AGC​CAT​AAA​ACC -3′), c region (F-5′- CCC​GCT​TTA​ATA​AGA​GAC​TTG​TGC​TCT​GCT​AAT​CGG​GGG​AGG -3′, R-5′- CCT​CCC​CCG​ATT​AGC​AGA​GCA​CAA​GTC​TCT​TAT​TAA​AGC​GGG -3′), and HCR lacking the b region (F-5′- TGG​CAA​GCC​CCC​CCT​CCT​TTT​GGG​GGT​TGC​GGG​AAA​TCC​T -3′, R-5′- AGG​ATT​TCC​CGC​AAC​CCC​CAA​AAG​GAG​GGG​GGG​CTT​GCC​A -3′).

### 2.3 Assessment of enhancer activity

Enhancer activity was assessed as previously described (Saito et al., 2019). Embryos were harvested and placed in PBS. Fluorescence was observed using a Leica M205FA fluorescence stereomicroscope (Leica, Germany) with the following bandpass filters: GFP3 (excitation at 450–490 nm, emission at 500–550 nm) and TXR (excitation at 540–580 nm, emission at 610 LP). Images were captured using a Leica DFC450C camera in RGB color mode (1,280 × 960 pixels, 2 × 2 Color Binning). For the quantification of fluorescence intensity, images were processed using ImageJ software (NIH, https://imagej.nih.gov/ij/index.html). The electroporated area was selected manually and cropped from the image captured using the GFP3 filter. The same areas were cropped from images captured using TXR filters. Subsequently, the images were split into red, green, and blue channels. Green and red channel images were used to quantify fluorescence intensity using GFP3 or TXR filters. Based on the fluorescence intensity measured using the GFP3 filter, the background intensity was subtracted and the area for measuring the fluorescence intensity was selected. The average fluorescence intensity within a selected area of each image was quantified.

### 2.4 Early chick (EC) culture

EC culture was performed according to a previously described protocol (Chapman et al., 2001), with some modifications. An HH 9 chick embryo was placed on the culture gel (0.15% glucose, 0.3% agarose, 62.5 mM NaCl, ovalbumin) with the dorsal side down. Then, 100 μL of the chemical such as activators and inhibitors diluted in Tyrode’s solution (8 g NaCl, 0.2 g KCl, 0.2 g CaCl_2_, 0.1 g MgCl_2_, 0.05 g NaH_2_PO_4_, 1 g glucose, 1 g NaHCO_3_, up to 1 L H_2_O) was added dropwise. The lid of the dish was closed and left at room temperature for approximately 1 h, and the dish was incubated at 38.6°C. The following chemical solutions were used: 1% DMSO (control), 1 μM PD173074 (Santa Cruz Biotechnology, TX, USA; sc-202610), 10 μM XAV939 (Cayman Chemical, MI, USA; 13,596), 20 μM CHIR-99021 (Focus Biomolecules, PA, USA; 10–1,279), 300 μM iCRT3 (Selleck, TX, USA; S8647), and 500 μM LF3 (Selleck, TX, USA; S8474), 10 μM U73122 (Cayman Chemical, MI, USA; 70,740), 100 μM LY294002 (FUJIFILM Wako, Japan; 129–04861), 50 μM Ruxolitinib (Selleck, TX, USA; S1378), 100 μM U0126 (Chemscene, NJ, USA; CS-0173).

### 2.5 Bead implantation

Heparin-coated acrylic beads (Sigma Aldrich, MO, USA; H5263) were added to 0.1% BSA/PBS (FUJIFILM Wako, Japan; 013–15104) solution or 100 ng/μL or 500 ng/μL FGF8b (R&D systems, MN, USA; 423-F8-025)/0.1% BSA solution and allowed to adsorb proteins for 1 h at room temperature. The beads were then implanted ventrally into the chick embryos and cultured on the culture gel described above.

### 2.6 Electroporation into whole embryos

We cracked the fertilized eggs at HH 4 into a glass dish and removed the albumin surrounding the embryos. We attached the embryo to the perforated paper and cut it around the paper and vitellin membrane. We transferred the embryo to warm Hank’s solution and removed the excess yolk. Embryos were placed dorsally on the anode in a dish (BEX, CUY701P2E) containing warm Hank’s solution. A DNA solution was injected between the embryo and the vitelline membrane. Embryos were sandwiched between platinum electrodes (BEX, CUY701P2L), and electric pulses (50 ms pulse on and 100 ms pulse off at 7 V, five times) were applied using a GEB14 electroporator (BEX). Embryos were washed with warm Hank’s solution and placed with ventral side up on the culture gel. Then, 50 μL yolk solution was added to the embryos, followed by incubation at 38.6°C. The yolk solution comprised fresh egg yolk and Hank’s solution, which were mixed in a 1:1 ratio and centrifuged, after which 0.5 mL of the supernatant was collected and mixed with 4.5 mL Hank’s solution.

### 2.7 Whole-mount *in situ* hybridization

Whole-mount *in situ* hybridization was conducted as previously described (Dietrich et al., 1997; Matsubara et al., 2017). Unless otherwise stated, shaking was performed in all steps. Collected embryos were fixed in 4% paraformaldehyde (PFA) in PBS overnight at 4 °C, without shaking. The embryos were then placed in 25% methanol in PBST (0.1% Tween 20 in PBS) and dehydrated for 10 min. Dehydration was performed by a stepwise increase in the concentration of methanol (50%, 75%, and 100% methanol, 10 min each step). Embryos were washed with 50% methanol in PBST for 10 min and twice with PBST for 5 min. The embryos were bleached in 6% H_2_O_2_ in PBST for 15 min. After washing twice with PBST for 5 min, the embryos were treated twice with detergent (1% NP-40, 1% SDS, 0.5% deoxycholate, 50 mM Tris pH 8.0, 1 mM EDTA, and 150 mM NaCl) for 20 min. Embryos were fixed in 0.2% glutaraldehyde in 4% PFA/PBS for 20 min. After washing twice with PBST for 5 min, the embryos were prehybridized in hybridization mix (50% formamide, 5× SSC pH 5.0, 250 μg/mL tRNA, 100 μg/mL heparin, 2% SDS) at 70 °C for 1 h. Then, a 1 μg/mL DIG-labeled probe was added to the hybridization mix, and embryos were incubated overnight at 70 °C. The next day, the embryos were washed four times for 30 min at 70 °C with wash solution (50% formamide, 2× SSC pH 5.0, 1% SDS) and then with 50% wash solution in TBST (0.1% Tween 20) at 70 °C for 10 min, followed by cooling to room temperature. After washing with TBST three times for 30 min, the embryos were blocked in 20% bovine serum in TBST for 1 h at 4 °C. Embryos were placed in 20% bovine serum in TBST containing 1:2000 Anti Digoxigenin-Ap Fab fragments (Roche, Switzerland; 11093274910), and the antibody reaction was performed overnight at 4 °C. The next day, after washing with TBST three times for 5 min and then seven times for 1 h, the embryos were washed overnight with TBST at 4 °C. The following day, the embryos were treated four times for 10 min with NTMT (100 mM NaCl, 100 mM Tris-HCl pH 9.5, 50 mM MgCl2, and 1% Tween 20), with levamisole added to a final concentration of 2 mM. Embryos were placed in NTMT containing 10% PVA, 3.5 μL/mL BCIP (Roche, 11383221001), and 3.5 μL/mL NBT (Roche, 11383213001), and color development was performed without shaking. After confirming sufficient coloring, the embryos were washed twice with TBST for 5 min to stop color development. Embryos were fixed in 4% PFA/PBS and stored at 4 °C. The DNA used as a template for synthesizing DIG-labeled RNA was amplified by PCR using the following primers: *mGdf11* (F-5′- GTA​TTA​AGC​CTC​CAG​GGT​TGG​GAA​T 3′, R-5′- CAG​GTG​TAT​ATT​CAT​AAG​ACA​ACC​CCT​TCC -3′), *mHoxd11* (Robert et al., 1995), and *cGdf11* (F-5′- AGC​AAA​GAG​CTG​CGG​CTG​GAG​AG -3′, R-5′- AAA​GGG​CAG​AAG​AGG​AGA​AGG​AAT​CCG​TC -3′).

### 2.8 Quantitative expression by RT-qPCR

Tissues were isolated from the pAM of chick embryos and posterior tissues containing the PSM of mouse embryos (E9.5). Whole embryonic bodies were harvested at E8.5. RNA was extracted from these tissues using the NucleoSpin RNA XS kit (TaKaRa, Japan; U0902B), and RNA was extracted from other tissues using TRIZOL Reagent (Invitrogen, 15596026). The Qubit RNA HS Assay Kit (Life Technologies, Carlsbad, CA, USA; Q32855) was used to measure the RNA concentration. RT-qPCR was performed using the One Step TB Green PrimeScript PLUS RT-PCR Kit (TaKaRa, RR096A) and StepOnePlus real-time PCR system (Life Technologies). The PCR cycle was 42 °C for 5 min; 95 °C for 10 s; 40 cycles of 95 °C for 5 s and 60 °C for 35 s; 95 °C for 15 s; 60 °C for 1 min; and 95 °C for 15 s. The following primers were used: *cGapdh* (F-5′- TCC​TCT​CTG​GCA​AAG​TCC​A -3′, R-5′- TCC​GTG​TGT​AGA​ATC​ATA​TTT​GAA​C -3′), *cGdf11* (F-5′- CAT​CGA​GAT​CAA​CGC​CTT​C -3′, R-5′- AGTCCAAGCCCAGGTTCC -3′), *mGapdh* (F-5′- TCA​CCA​CCA​TGG​AGA​AGG​C -3′, R-5′- GCT​AAG​CAG​TTG​GTG​GTG​CA -3′), and *mGdf11* (F-5′- CAC​AGA​CCT​GGC​TGT​CAC​C -3′, R-5′- TCG​AAG​CTC​CAT​GAA​AGG​AT -3′).

### 2.9 Preparation of cryosections

The collected embryos were fixed in 4% PFA in PBS on ice for 30 min. After trimming the embryos, the tissues were dehydrated in 10% sucrose in PBS at 4 °C for 1 h. They were then dehydrated stepwise with 20% sucrose in PBS and 30% sucrose in PBS at 4 °C for 1 h. Tissues were embedded using Frozen Section Compound (Leica, 3801481) and stored at −80°C. Tissues were sectioned at 20 μm thickness using a cryostat (Leica, CM3050). The sections were placed on a MAS-coated glass slide (MATSUNAMI, Japan; S9115) and dried using a slide warmer at 40 °C for 1 h. The glass slides were washed three times with PBS for 1 min. After treatment with 0.1% TritonX-100 in PBS and 1 μg/mL DAPI at room temperature for 10 min, the glass slide was washed with PBS three times for 1 min. The slides were mounted in Fluorescence Mounting Medium (Dako, Agilent, CA, USA; S3023). Fluorescence was observed under the Leica DM2500 fluorescence microscope.

### 2.10 Genome editing

Cas9 protein, two crRNAs flanking the coding sequence or enhancers of *Gdf11*, and tracrRNA were introduced into in vitro-fertilized zygotes by electroporation (GEEP method), as reported (Hashimoto and Takemoto, 2015). The target sequences were as follows: *Gdf11* crRNA1:5-GGAGCCGTAAACAAGCCAAG-3, *Gdf11* crRNA2:5-TTCTACCAGACCATAACTGC-3, HCR crRNA1:5-ACTCAGGCTGGATGGCCCAG-3, HCR crRNA2:5-GAGTTGCTGAGACGTACACC -3. Electroporated embryos were transferred into the oviducts of female pseudopregnant B6D2F1 (BDF1) mice. Chimeric mice were crossed with B6D2F1 (BDF1) mice purchased from Japan SLC, and the strain was established. Deletion of the *Gdf11* coding sequence or HCR was confirmed by PCR amplification of the target genomic region and sequencing. Cas9 protein, crRNA, and tracrRNA were purchased from IDT (IA, USA).

### 2.11 Genotyping

Tissue obtained by ear punching was placed in lysis buffer (10 mM Tris-HCl pH 8.3, 50 mM KCl, 2.5 mM MgCl_2_, 0.45% NP40, 0.45% Tween 20, 200 μg/μL Proteinase K) and lysed overnight at 56 °C. After extracting genomic DNA, genotyping was performed under the following conditions. To evaluate the genotype of HCR-deficient mice, the following primers were used: wild-type allele (F-5′- ATA​GTG​AAG​GCA​ATG​GGA​AGC​CTG -3′, R-5′- TTG​ATT​GAC​TAA​GGG​CAG​GGA​TAG​G -3′), *∆HCR* allele (F-5′- GGG​CCA​CAT​CTG​TGT​TGG​ATT​G -3′, R-5′- ACC​AGG​CAG​GTT​GTG​AGC​TAT​TG -3′). To evaluate the genotype of *Gdf11*-deficient mice, the following primers were used: wild-type allele (F-5′- AGA​TTA​TCT​ACG​GCA​AGA​TCC​CTG​G -3′, R-5′- TGT​TGT​ATT​GCA​CAC​TGC​TTG​GTC -3′) and *Gdf11*-deficient allele (F-5′- CCA​GGA​GCT​CTA​GAC​CGT​TAC​C -3′, R-5′- GCT​TTT​CTG​TTC​CTC​TCC​TAC​ACC -3′).

The PCR cycle was 98 °C for 2 min; 35 cycles of 98 °C for 10 s, 60 °C for 30 s, and 68 °C for 30 s; and 68 °C for 1 min.

### 2.12 Skeletal staining

P0 mice were harvested and stored at −30°C. The frozen mice were placed in hot water at approximately 37 °C and completely thawed. The mice were placed in hot water (60 °C) for approximately 1 min until the surface became white and then transferred to distilled water at room temperature. The raised epidermis of the mice was peeled off in distilled water, and the abdomen was opened. Mice were dehydrated and fixed in 90% ethanol for 15 min. The skin, fat, and internal organs of the mice were removed, placed in 90% ethanol, and stored overnight in 99.5% ethanol at 4 °C. Unless otherwise stated, the following steps were performed under shaking. Mice were placed in acetone overnight and then in 0.015% Alcian blue (Sigma-Aldrich, A5268) in 20% acetic acid/80% ethanol solution for two nights. After shaking twice for 1 h in 95% ethanol, the mice were placed in 0.0125% alizarin red (FUJIFILM Wako, 011–01192) in 70% ethanol for 2 h. Mice were placed in 1% KOH and incubated overnight at 4 °C. Subsequently, KOH was added without shaking. Mice were placed in 0.8% KOH in 20% glycerol until the tissues became transparent and then in 0.6% KOH in 40% glycerol overnight. Mice were shaken overnight in 0.4% KOH in 60% glycerol and then placed in 0.2% KOH in 80% glycerol overnight. The mice were placed in 100% glycerol and stored. The skeletal patterns were observed and photographed using a stereomicroscope (Leica M205FA) and camera (Leica DFC450C).

## 3 Results

### 3.1 Identification of three conserved regions surrounding the *Gdf11* locus

**FIGURE 1:**
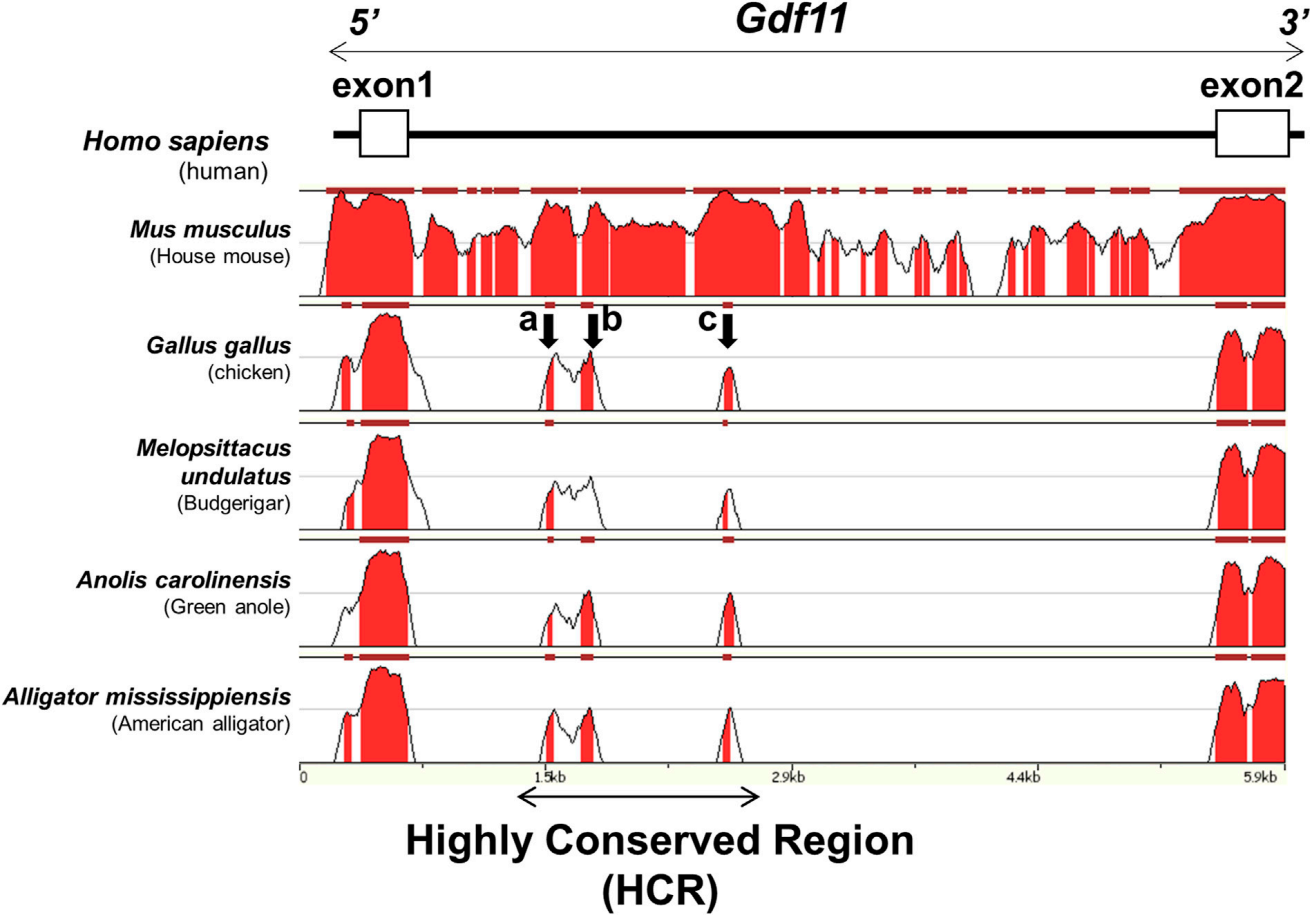
Three conserved regions surrounding the *Gdf11* loci Conserved regions involving intron 1 of human *Gdf11* (hg38, chr12:55743200–55749100) among tetrapod species. The regions with the peaks were conserved. Three regions of approximately 50 bp (a, b, and c), indicated by arrows, were highly conserved. A region of approximately 1700 bp including all three regions, was named HCR.

To clarify the molecular mechanisms that induce *Gdf11* expression, we tried to identify candidate enhancer sequences that regulate the expression of *Gdf11 in silico*. We searched for conserved sequences in noncoding regions within 30 kb of the human *Gdf11* locus (hg38, chr12:55728000–55758000) across the species human, mouse, chicken, budgerigar, green anole, and American alligator, using the ECR genome browser. Conservation of the noncoding region surrounding the *Gdf11* locus was very low; therefore, we did not detect any putative conserved regions either upstream or downstream of the *Gdf11* gene. We found only three highly conserved regions in intron 1 of the *Gdf11* gene. We named this region the Highly Conserved Region (HCR) (1727 bp; mm9, chr10:128326106-128327832), and three regions, including the HCR, a, b, and c regions (Figure 1).

### 3.2 The HCR functions as a *Gdf11* enhancer and is essential for positioning of the sacral-hindlimb unit

**FIGURE 2:**
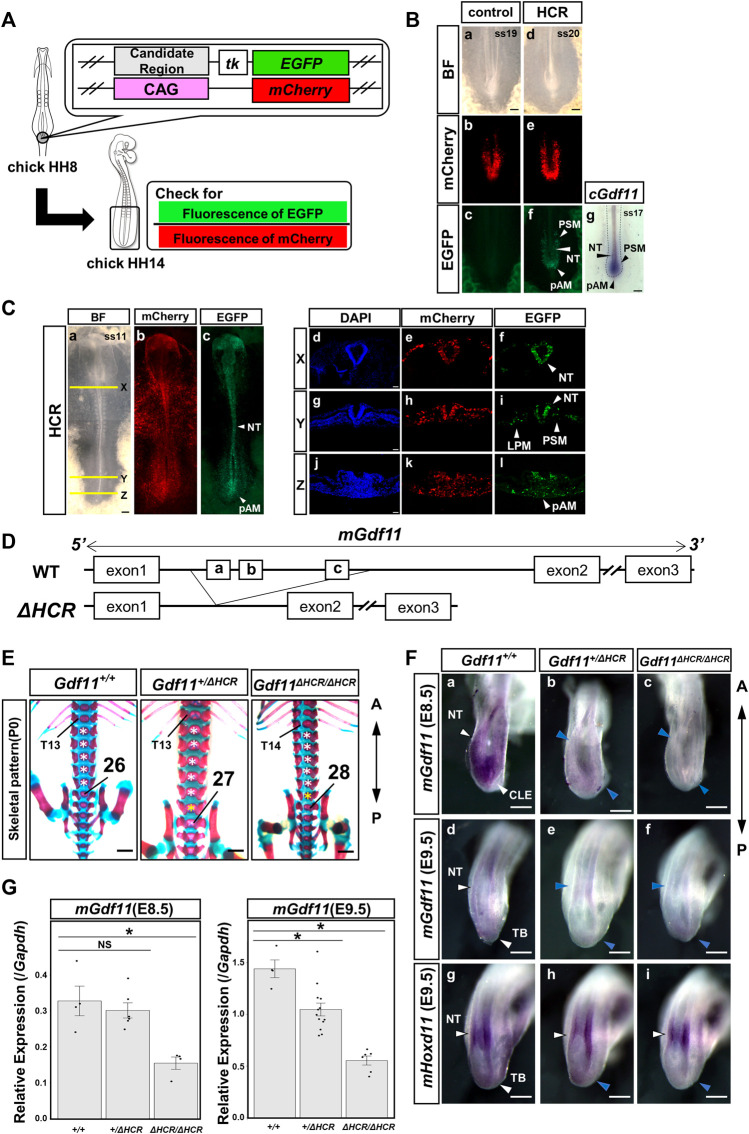
The HCR functions as a *Gdf11* enhancer and is essential for positioning of the sacral-hindlimb unit. **(A)** Schematic diagram of a reporter assay using chick embryos. **(B)** Reporter assay results for controls **(a–c)** and HCR **(d–f)**. HCR exhibited enhanced activity in the pAM, PSM, and NT. Number of EGFP-positive samples/number of all examined samples: 0/8 control; 9/9 HCR. **(g)** Endogenous *cGdf11* expression by *in situ* hybridization. ss: somite stage. Scale bar: 200 μm. **(C)** Gene transfer into whole embryos via electroporation. HCR exhibits enhanced activity simultaneously with *Gdf11* expression. Whole embryo **(a–c)** and tissue section images (X: d-f, Y: g-I, Z: j-l). Number of EGFP-positive samples/total number of examined samples: 12/12; HCR. Scale bar: 200 μm **(a–c)**, 50 μm (d–l). **(D)** Method of generating HCR-deficient mice. Wild-type (top) and HCR-deficient mice (bottom). **(E)** Phenotypic analysis of P0 wild-type mice and mice deficient in the HCR (*Gdf11* ^*+/ΔHCR*^, *Gdf11* ^*ΔHCR/ΔHCR*^). T, thoracic; white asterisks, normal number of lumbar vertebrae; yellow asterisks, extra lumbar vertebrae. Scale bar: 1 mm. **(F)** Expression analysis of *Gdf11* and its downstream target *Hoxd11* in wild-type mice and *Gdf11* ^*+/ΔHCR*^, *Gdf11* ^*ΔHCR/ΔHCR*^ mice at E8.5 (a–c) and E9.5 (d–i). Arrowheads indicate gene expression regions. Blue arrowheads indicate decreased expression levels. NT, neural tube; CLE, caudal lateral epiblast; TB, tail bud Scale bar: 200 μm. **(G)** Expression level of *Gdf11* at E8.5 and E9.5 in wild-type mice and *Gdf11* ^*+/ΔHCR*^, *Gdf11* ^*ΔHCR/ΔHCR*^ mice by RT-qPCR. *p < 0.05 (Steel test). Dots indicate the relative expression levels of *Gdf11* in each sample. Error bars indicate standard error of the mean.

To investigate whether HCR displayed enhancer activity in PSM, we performed a reporter assay using *in ovo* electroporation. The *EGFP* reporter fused to the mouse HCR was electroporated together with pCAGGS-*mCherry* (for visualization of the electroporated region) in the posterior region of Hensen’s node in HH 8 chick embryos (Figure 2A). We observed a strong EGFP signal in the pAM, caudal neural tube (NT), and PSM (9/9) (Figures 2Ba-f), where *Gdf11* was expressed (Figures 2Bg). This result suggests that the HCR contains *Gdf11* enhancers during this developmental stage.

To further confirm that the enhancer activity of HCR was activated in the embryo, we electroporated the reporter constructs into whole embryos and observed EGFP fluorescence (Figure 2C). We observed EGFP fluorescence in the neural tissue from the brain to the posterior NT, whereas in the mesodermal tissue, EGFP fluorescence was confined to the PSM and LPM in the posterior region, with weak expression (12/12) (Figures 2Ca–c). We further analyzed the embryonic tissue sections (Figures 2Cd–l). EGFP fluorescence was observed in the pAM, posterior PSM, NT, and LPM. These results indicate that HCR possesses enhancer activity that extends beyond the endogenous *Gdf11-*expressing region.

Since the HCR showed enhancer activity, we generated HCR-knockout (*Gdf11* ^*ΔHCR/ΔHCR*^) mice and analyzed the skeletal pattern and endogenous *Gdf11* expression (Figure 2D). Compared with wild-type mice (*Gdf11* ^*+/+*^), *Gdf11* ^*+/ΔHCR*^ mice had one extra thoracic (C7T14L5S4) or lumbar (C7T13L6S4) vertebra, and the position of the sacral-hindlimb unit was shifted posteriorly (Figure 2E; Table 1, Supplementary Figure S1). The number of thoracic and lumbar vertebrae was increased by one (C7T14L6S4) in *Gdf11* ^*ΔHCR/ΔHCR*^ mice, which shifted the position of the sacral-hindlimb unit posteriorly by up to two vertebrae (Figure 2E; Table 1, Supplementary Figure S1). Therefore, the HCR is essential for positioning the sacral-hindlimb unit.

**TABLE 1:** Skeletal analysis of wild-type, *Gdf11^+/ΔHCR^*, and *Gdf11^ΔHCR/ΔHCR^* mice.

	n = 21	n = 33	n = 22
	*Gdf11* ^*+/+*^	*Gdf11^+/ΔHCR^*	*Gdf11^ΔHCR/ΔHCR^*
Presacral vertebrae			
25	13	1	
26	8	32	20
27			2
Vertebral pattern			
C7 T13 L5	13	1	
C7 T13 L6	8	22	6
C7 T14 L5		10	14
C7 T14 L6			2
Attached/unattached ribs			
7/6	21	23	6
7/7		7	
8/6		3	16

C: cervical, T: thoracic, L: lumber.

Next, to investigate changes in endogenous mouse *Gdf11* (*mGdf11*) expression, we analyzed embryos at E8.5 (*mGdf11* expression onset) and E9.5, by whole-mount *in situ* hybridization and RT-qPCR. In E8.5 embryos, endogenous *mGdf11* expression level at the onset of expression in *Gdf11* ^*ΔHCR/ΔHCR*^ mice was though remarkably reduced in the posterior NT and CLE (Figures 2Fa–c), it was still expressed. Next, we analyzed the expression level of *mGdf11* in E8.5 embryos quantitatively by RT-qPCR. The expression level of endogenous *mGdf11* in *Gdf11* ^*ΔHCR/ΔHCR*^ mice was significantly reduced, to approximately 40% of that in the wild-type mice (Figure 2G). At E9.5, we analyzed the expression of *mGdf11* and *mHoxd11*, a gene downstream of *Gdf11*. In *Gdf11* ^*ΔHCR/ΔHCR*^ embryos, the expression level of *mGdf11* was decreased in the NT and especially in the tailbud, and anterior domain of *mHoxd11* expression in NT and LPM was shifted posteriorly and its expression of the tailbud was reduced (Figures 2Fd-i). The expression level of endogenous *mGdf11* in *Gdf11* ^*+/ΔHCR*^ and *Gdf11* ^*ΔHCR/ΔHCR*^ mice in E9.5 embryos was significantly reduced, to approximately 70% and 30% of those in the wild-type mice, respectively (Figure 2G). These results indicate that the HCR is essential for determining the position of the sacral-hindlimb unit, and that the *Gdf11* enhancer exists within this region.

### 3.3 Only b region shows enhancer activity among the conserved individual regions, a, b, and c

**FIGURE 3:**
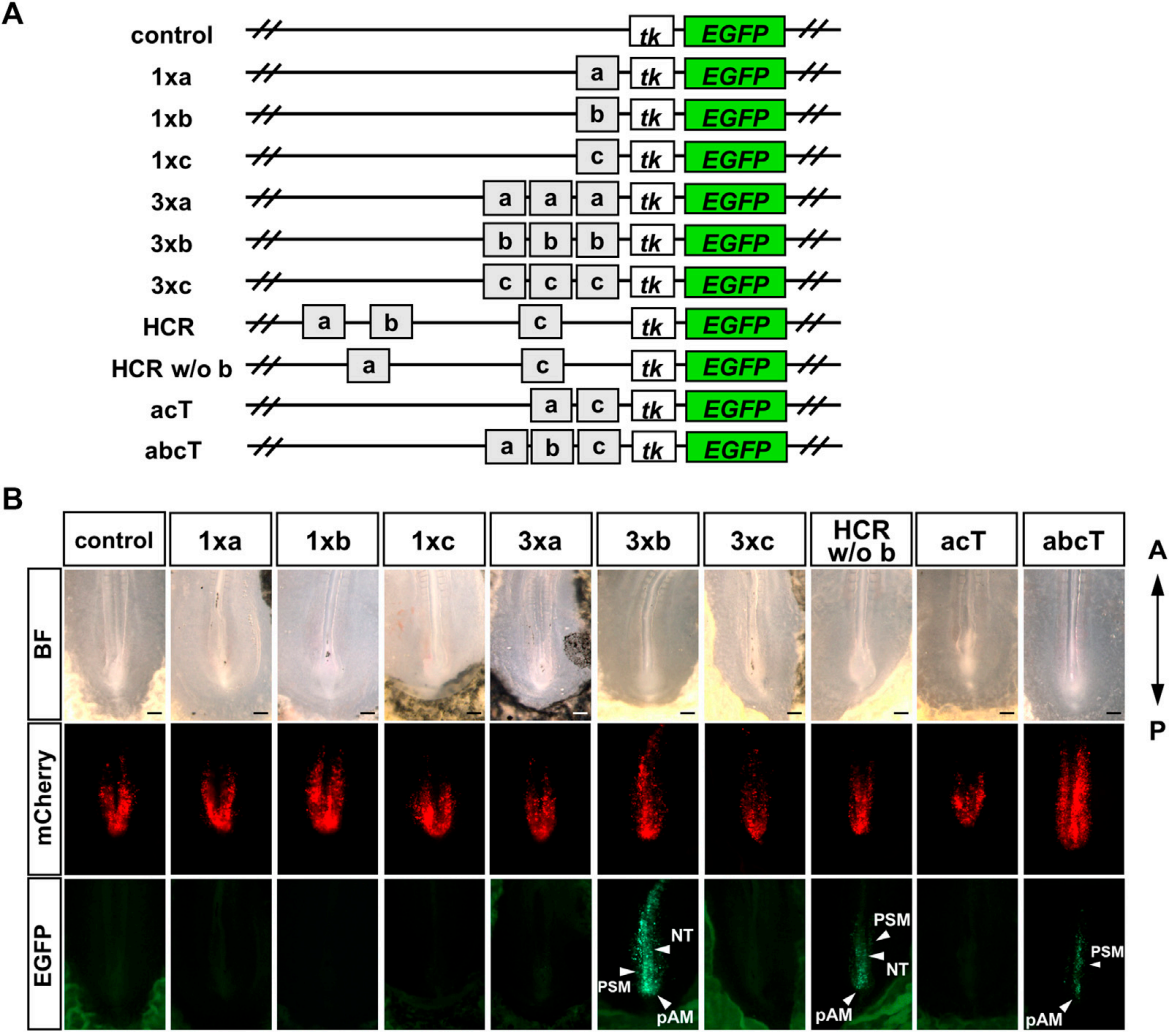
Only b region shows enhancer activity among the conserved individual regions, (a, b, c) **(A)** Schematic representation of the analysis of conserved regions in *Gdf11* intron 1. We constructed an EGFP reporter in which single a, b, and c regions were inserted upstream of *tk-EGFP* (1xa, 1xb, 1xc). Each a, b, and c region was repeated in tandem three times (3xa, 3xb, 3xc), only the b region was deleted from the HCR (HCR w/o b). The a and c regions were tandemly connected (acT), and the separate a, b, and c regions were tandemly connected (abcT). Number of EGFP-positive samples/total number of examined samples: 0/8, control; 0/5, 1xa; 0/11, 1xb; 0/3, 1xc; 0/6, 3xa; 7/7, 3xb; 0/5, 3xc; 8/8, HCR w/o b; 0/10, acT; 12/18, abcT. Scale bar: 200 μm. Arrowheads: regions with enhancer activity. BF, bright field; NT, neural tube; PSM, presomitic mesoderm; pAM, posterior axial mesoderm.

Next, we investigated which conserved sequences within the HCR contribute to enhancer activity. We constructed an EGFP reporter in which single a, b, and c regions were inserted upstream of *tk-EGFP* (1xa, 1xb, 1xc); each a, b, and c region was tandemly repeated three times (3xa, 3xb, 3xc). The separate a, b, and c regions were tandemly connected (abcT) and the a and c regions were tandemly connected (acT) (Figure 3A). We then performed a reporter assay in chick embryos. EGFP fluorescence was not detected in the embryos electroporated with 1xa, 1xb, 1xc. Next, we electroporated with 3xa, 3xb, 3xc constructs with the anticipation that each enhancer activity would be more readily observable. In embryos electroporated with 3xb; strong EGFP fluorescence was observed in the pAM, PSM, and NT, whereas no significant EGFP fluorescence was observed in embryos electroporated with 3xa and 3xc. These results suggest that b region may be important for HCR enhancer activity. Therefore, we prepared a sequence in which only the b region was deleted from the HCR (HCR w/o b). We observed EGFP fluorescence in the pAM, PSM, and NT in embryos electroporated with HCR w/o b (Figure 3B). These findings imply that the HCR contains sequences other than the b region that are necessary for its enhancer function. Next, we prepared a sequence in which only the a- and c-regions were connected (acT). EGFP fluorescence was not observed in embryos electroporated with the acT construct (Figure 3B). When we electroporated the abcT construct, we observed EGFP fluorescence in the PSM and pAM; however, this fluorescence was weaker than that observed in embryos electroporated with HCR (Figure 3B). These results suggest that the b region is important for enhancer activity among the conserved individual regions (a, b, and c); however, HCR contains non-conserved sequences that can compensate for the enhancer activity of the b region.

### 3.4 *Gdf11* ^*ΔHCR/-*^ mice exhibit a more severe phenotype compared with *Gdf11* ^*ΔHCR/ΔHCR*^ and *Gdf11* ^*+/−*^ mice

**FIGURE 4:**
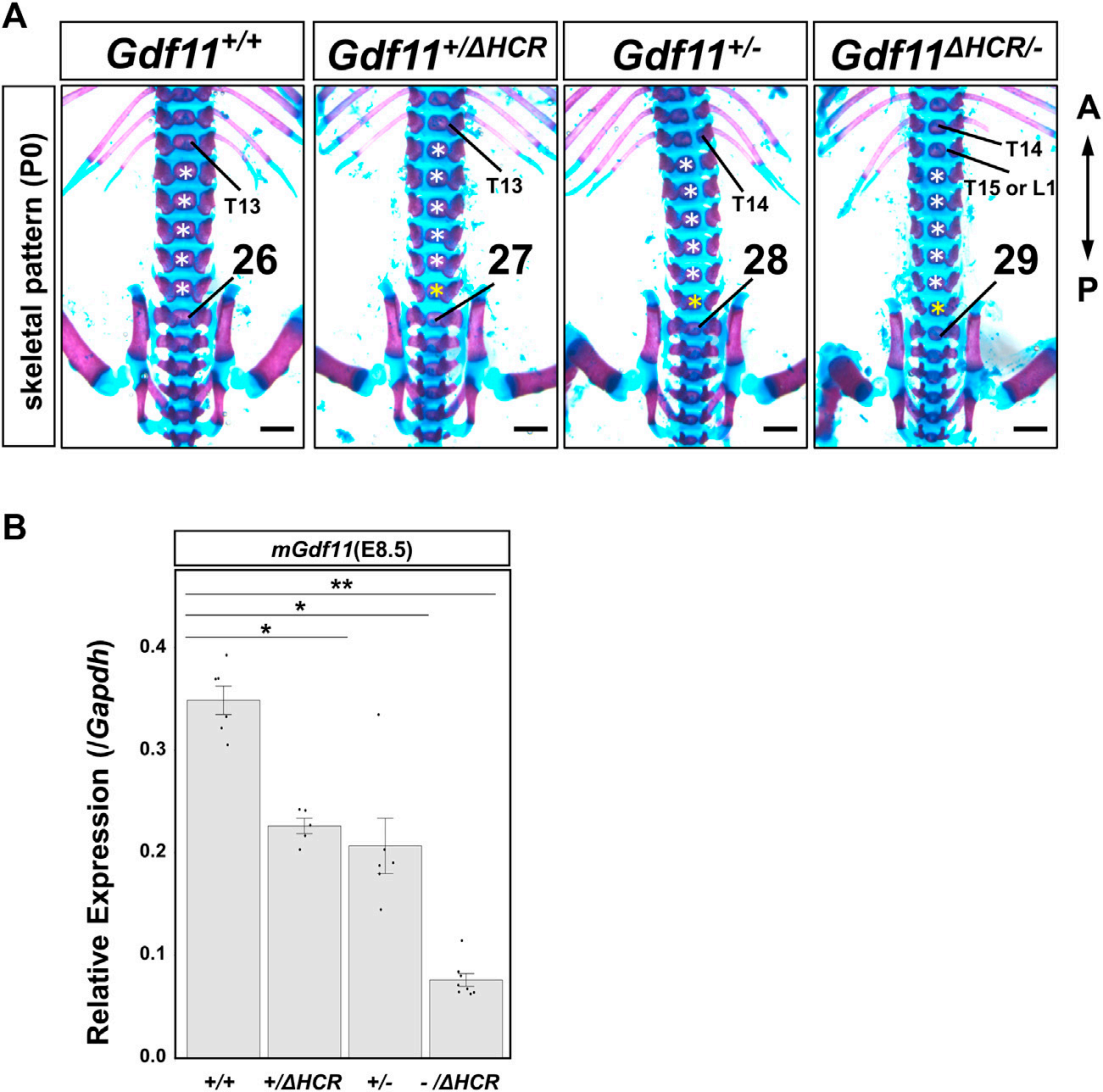
The position of the sacral hindlimb unit was shifted more posteriorly in *Gdf11* ^*ΔHCR/-*^ mice. **(A)** Phenotypic analysis of *Gdf11* ^*+/+*^, *Gdf11* ^*+/ΔHCR*^, *Gdf11* ^*+/−*^, and *Gdf11* ^*ΔHCR/−*^ mice at P0. *Gdf11* ^*ΔHCR/−*^ mice showed posterior displacement of the first sacral vertebrae by two to three vertebrae. T: thoracic; L: lumbar. White asterisks: normal number of lumbar vertebrae; yellow asterisks: extra lumbar vertebrae. Scale bar: 1 mm. **(B)** Expression level of *Gdf11* at E8.5 in *Gdf11* ^*+/+*^, *Gdf11* ^*+/ΔHCR*^, *Gdf11* ^*+/−*^, and *Gdf11* ^*ΔHCR/−*^ mice by RT-qPCR. *p < 0.05, **p < 0.01 (Steel test). Dots indicate the relative expression levels of *Gdf11* in each sample. Error bars indicate standard error of the mean.

Based on our results, we predicted that the position of the sacral-hindlimb unit is determined by the expression level of *Gdf11*. Therefore, we generated transheterozygous *Gdf11* mice by crossing *Gdf11* ^*+/−*^ and *Gdf11* ^*+/ΔHCR*^ mice (Figure 4A; Table 2, Supplementary Figure S2). As a result, the position of the sacral-hindlimb unit was shifted posteriorly by up to three vertebrae in *Gdf11* ^*ΔHCR/−*^ mice due to the addition of two thoracic vertebrae and one extra lumbar vertebra (C7T15L6S4). Next, we analyzed the expression level of *mGdf11* in E8.5, using RT-qPCR. The expression level of endogenous *mGdf11* in *Gdf11* ^*ΔHCR/-*^ was significantly reduced, to approximately 20% of that in the wild-type mice (Figure 4B). These results indicate that *Gdf11* ^*ΔHCR/-*^ mice show more severe phenotype than that of *Gdf11* ^*+/−*^ and *Gdf11* ^*ΔHCR/ΔHCR*^ mice, depending on expression level of *Gdf11*.

**TABLE 2:** Skeletal analysis of wild-type, *Gdf11^+/ΔHCR^*, *Gdf11^+/−^*, and *Gdf11^ΔHCR/-^* mice.

	n = 7	n = 9	n = 8	n = 6
	*Gdf11* ^*+/+*^	*Gdf11^+/ΔHCR^*	*Gdf11* ^*+/−*^	*Gdf11^ΔHCR/-^*
Presacral vertebrae				
25	3			
25/26	1			
26	3	6		1
26/27			1	
27		2	7	1
27/28				1
28		1		3
Vertebral pattern				
C7 T13 L5	3			
C7 T13 L6	3	3		
C7 T13 L5/6	1			
C7 T14 L5		5		1
C7 T14 L5/6			1	
C7 T14 L6			7	1
C7 T14 T/L1 L6				1
C7 T15 L6		1		3
Attached/unattached ribs				
7/6	7	2		
7/7		1		
8/5		1		
8/6		4	8	3
9/6		1		3

C: cervical, T: thoracic, L: lumber.

L5/6 or L6/7: on the one hand, the sacral rib is connected to the pelvis, and on the other, it is free from the pelvis. T/L1: one side has ribs and is shaped like a thoracic vertebra; the other side has no ribs and is shaped like a lumbar vertebra.

### 3.5 Inhibition of FGF signaling upregulated endogenous *Gdf11* expression

**FIGURE 5:**
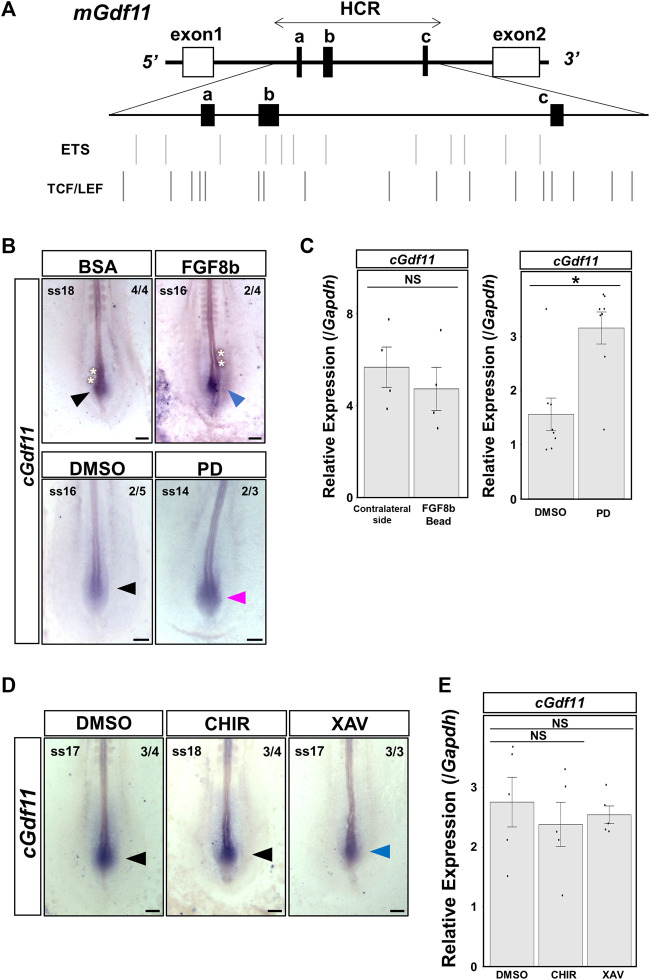
Inhibition of FGF signaling upregulated endogenous *Gdf11* expression. Endogenous *cGdf11* expression after FGF and WNT signaling was altered in chick embryos. **(A)** ETS transcription factor binding sites (TFBSs) and TCF4 and LEF1 TFBSs within the HCR of *mGdf11*. Bars indicate the TFBS positions. **(B, C)** *cGdf11* expression after altering FGF signaling levels. **(B)** *In situ* hybridization after treatment with 100 ng/μL FGF8b (top) or 1 μM PD173074 (bottom panel). The image with the strongest signal intensity obtained under the same conditions is shown. Representative signal intensities as pictured/all samples: 4/4, BSA; 2/4, FGF8b; 2/5, DMSO; 2/3, PD. FGF8b: 100 ng/μL FGF8b; PD: 1 μM PD173074 **(C)** RT-qPCR analysis after altering the FGF signaling levels. Implantation of 500 ng/μL FGF8b soaked beads and treatment of 1 μM PD173074 (right). *p < 0.05 (Mann-Whitney *U* test). Dots indicate the relative expression levels of *cGdf11* in each sample. Error bars indicate standard error of the mean. Bead: transplanted side; Opposite: non-transplanted side. **(D, E)** *cGdf11* expression after altering the WNT signaling levels. **(D)** *In situ* hybridization after treatment with DMSO (left), 20 μM CHIR-99021 (middle), or 10 μM XAV939 (right). Representative signal intensity as pictured/all samples: 3/4 DMSO; 3/4 CHIR; and 3/3 XAV. CHIR: 20 μM CHIR-99021; XAV: 10 μM XAV939 **(E)** RT-qPCR analysis after altering the WNT signaling levels. Dots indicate the relative expression levels of *cGdf11* in each sample. Error bars indicate standard error of the mean (Steel test). Scale bar: 200 μm. Arrowheads indicate the gene expression regions. The blue and red arrowheads indicate decreased and increased expression levels, respectively.

We used the rVista (https://rvista.dcode.org/) and JASPAR programs (https://jaspar.elixir.no/) to search for candidate upstream factors regulating *Gdf11* in the HCR. We found predicted ETS transcription factor binding sites (TFBSs) downstream of FGF signaling and TCF4 and LEF1 TFBSs downstream of WNT signaling (Figure 5A). Therefore, we focused on FGF and WNT signaling because they are essential for posterior elongation of the embryo. We analyzed the changes in endogenous *Gdf11* expression and enhancer activity in HCR after altering FGF and WNT signaling levels in chick embryos.

First, we transplanted 100 ng/μL FGF8b-soaked beads near the *Gdf11-*expressing region. Some individuals showed reduced expression of chicken *Gdf11* (*cGdf11*) on the side implanted with FGF8b beads (Figure 5B). Next, when we administered 1 μM PD173074 to inhibit FGF signaling, *cGdf11* expression was upregulated without affecting its expression region (Figure 5B). Furthermore, we quantitatively analyzed the expression levels of endogenous *cGdf11* (Figure 5C). When we implanted FGF8b beads on the right side of the pAM (FGF8b bead in Figure 5C), the expression level of *cGdf11* appeared to be downregulated compared to that on the left side of the pAM (contralateral side in Figure 5C); however, there was no statistically significant difference. In contrast, *cGdf11* expression significantly increased after treatment with 1 μM PD173074. These results suggest that FGF signaling negatively regulates endogenous *Gdf11* expression.

Next, we analyzed the changes in the expression of *cGdf11* after enhancement or inhibition of WNT signaling (Figures 5D,E). We did not observe a remarkable change in *cGdf11* expression after treatment with 20 μM CHIR-99021, an activator of WNT signaling (Figures 5D,E). On the other hand, treatment with 10 μM XAV939, a WNT signaling inhibitor, induced a slight decrease in *cGdf11* expression (Figure 5D), which was not statistically different as per RT-qPCR (Figure 5E). These results indicate that WNT signaling is not a major factor controlling endogenous *Gdf11* expression.

### 3.6 Upregulation of WNT signaling suppressed the enhancer activity of the HCR

To investigate whether FGF or WNT signaling regulates the enhancer activity of HCR, we performed a reporter assay and quantitatively analyzed the enhancer activity of HCR, based on our previous method (Saito et al., 2019). We used *EGFP* reporter fused to HCR and pCAGGS-*mCherry,* and calculated the relative intensity of EGFP to mCherry as an index of enhancer activity (Section 2.3) (Saito et al., 2019).

**FIGURE 6:**
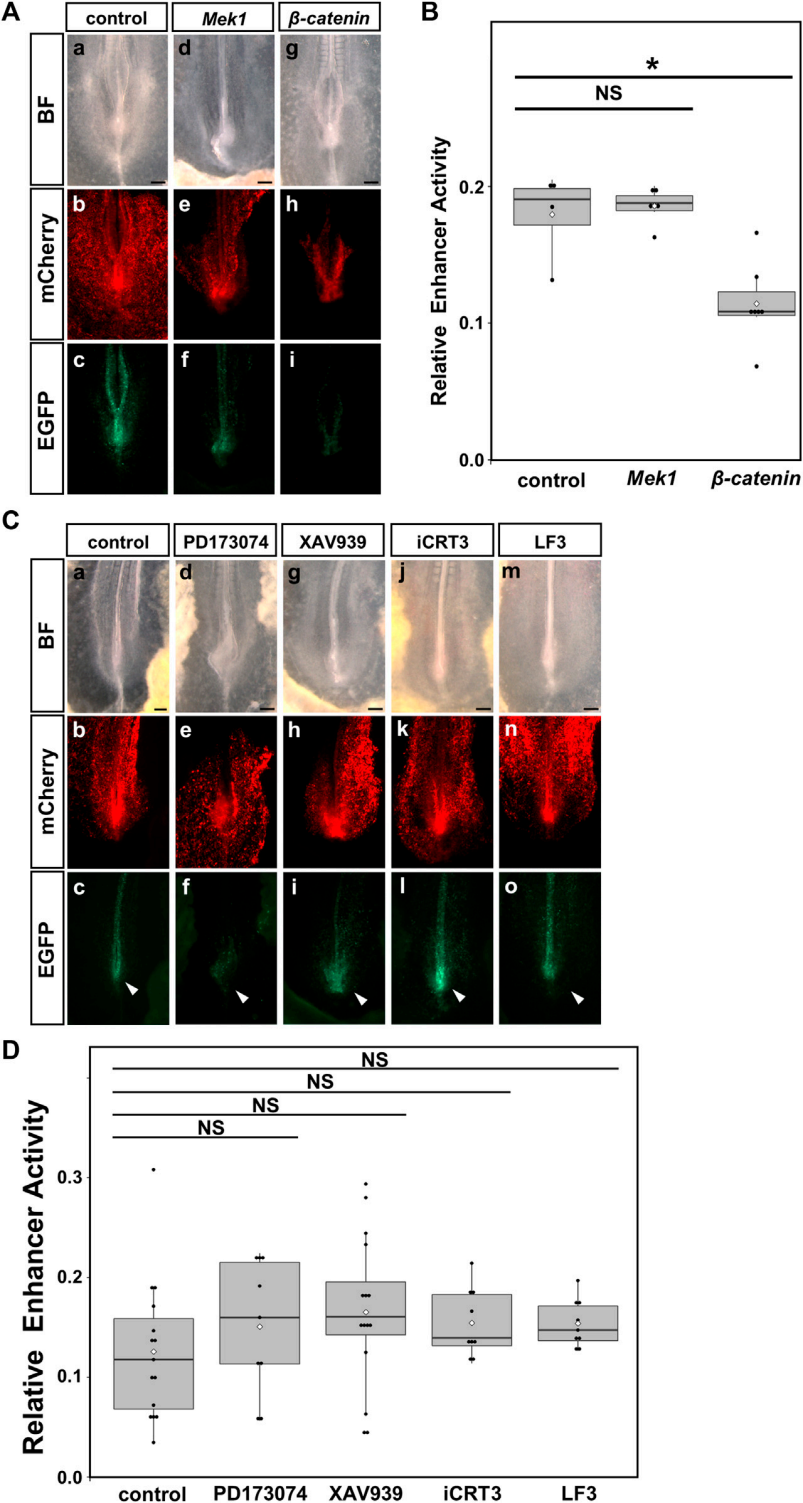
Upregulation of WNT signaling suppressed the enhancer activity of the HCR in the PSM. The enhanced activity of HCR after FGF and WNT signaling was altered in chick embryos. **(A)** Reporter assay for the control (left, (a–c)), overexpression of CA-*Mek1* (middle, (d–f)), overexpression of CA-*β-catenin* (right, (g–i)). **(B)** Quantification of enhancer activity in the HCR after overexpression of CA-*Mek1* and CA-*β-catenin*. Dots indicate the enhancer activity of each sample. Error bars indicate standard error of the mean. *p < 0.05 (Steel test). **(C)** Reporter assay for DMSO as control (a–c), 1 μM PD173074 (d–f), 10 μM XAV939 (g–i), 300 μM iCRT3 (j–l), 500 μM LF3 (m–o). **(D)** Quantification of enhancer activity in the HCR after treatment with 1 μM PD173074, 10 μM XAV939, 300 μM iCRT3 or 500 μM LF3. Dots indicate the enhancer activity of each sample. Error bars indicate standard error of the mean. BF: bright field, *Mek1*: CA-*Mek1*, *β-catenin*: CA-*β-catenin*, PD: 1 μM PD173074, XAV: 10 μM XAV939, iCRT3:300 μM iCRT3, LF3:500 μM LF3.

When we co-electroporated constitutively active *Mek1* (CA-*Mek1*), the enhancer activity of HCR did not change. On the other hand, the enhancer activity of the HCR was significantly reduced by constitutively active *β-catenin* (CA-*β-catenin*) co-electroporation (Figures 6A,B). Next, when we inhibited FGF signaling by 1 μM PD173074 treatment or WNT signaling by 10 μM XAV939, 300 μM iCRT3, 500 μM LF3 treatment, enhancer activity of HCR appeared to be slightly increased, however, they were not statistically different (Figures 6C,D). These results suggest that the upregulation of WNT signaling inhibits the enhancer activity of HCR; however, inhibition of endogenous FGF or WNT signaling does not significantly affect this enhancer activity.

## 4 Discussion

**FIGURE 7:**
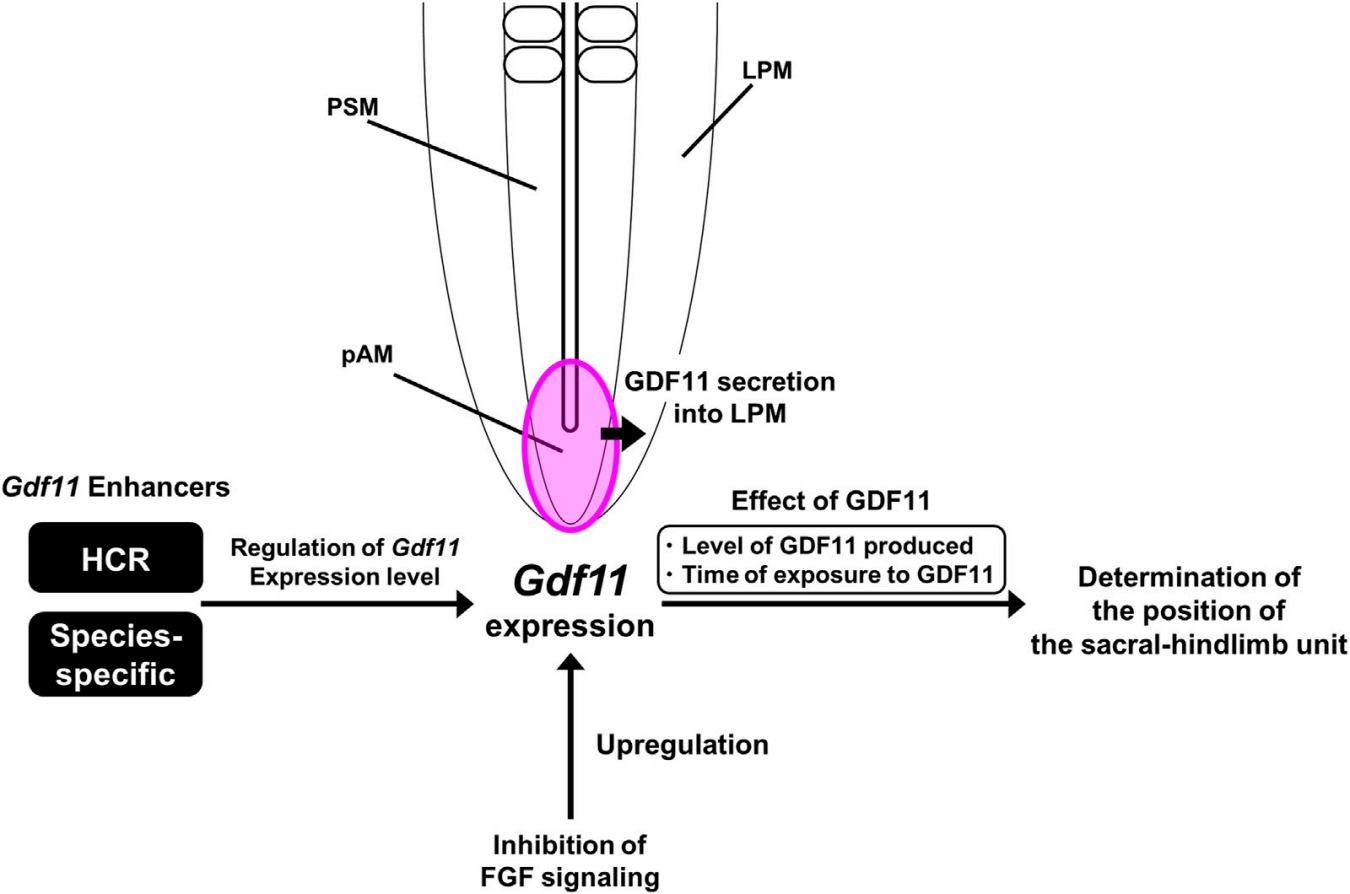
A model of *Gdf11* expression regulation. HCR regulates the level of *Gdf11* expression rather than the initiation timing of *Gdf11* expression. The phenotype of *Gdf11ΔHCR/ΔHCR* mice fails to mimic the phenotype of *Gdf11* ^−/−^ mice, suggesting the existence of more *Gdf11* enhancers other than the HCR. Because there are only three conserved regions around the *Gdf11* locus, the presence of species-specific *Gdf11* enhancers other than the three conserved regions (a‐c) was predicted. The position of the sacral hindlimb unit is thought to be determined by both the quantity of GDF11 produced and the duration of exposure to GDF11. Inhibition of FGF signaling upregulates the expression of endogenous *Gdf11* without mediating HCR.

In the present study, we found that only three regions (a, b, and c) among tetrapods, surrounding the *Gdf11* loci, were highly conserved. HCR showed enhancer activity in the pAM, in which endogenous *Gdf11* is expressed, and is essential for determining the position of the sacral-hindlimb unit as an enhancer of *Gdf11*. Among the three conserved regions, only region b exhibited enhancer activity. However, HCR, excluding region b, still showed enhancer activity. These results suggest that the b region is important for enhancer activity among the three conserved regions; however, HCR contains non-conserved sequences that can compensate for the enhancer activity of the b region. Determining whether the sacral-hindlimb unit and endogenous *Gdf11* expression undergo changes in mice lacking only the b region is an important question to address in the future. Furthermore, we found that inhibition of FGF signaling induced the upregulation of endogenous *Gdf11* expression. FGF signaling primarily activates the expression of target genes. Therefore, we predicted that the upregulation of endogenous *Gdf11* expression by the inhibition of FGF signaling is an indirect effect that does not mediate *Gdf11* enhancers, including HCR (Figure 7). To further identify the signaling cascade downstream of the FGF receptor that regulates *Gdf11* expression, we analyzed *Gdf11* expression by inhibiting individual pathways downstream of FGF signaling using specific inhibitors (U73122: PLC inhibitor, LY294002: PI3K inhibitor, Ruxolitinib: JAK1/2 inhibitor, and U0126: MEK1/2 inhibitor). Despite treatment with each inhibitor, no remarkable alteration was observed in *Gdf11* expression (Supplementary Figure S3). Therefore, it is likely that the cooperative function of all downstream signaling pathways of the FGF regulates endogenous *Gdf11* expression. Because FGF signaling is activated in Hensen’s nodes and primitive streaks earlier than *Gdf11* expression onset (Chapman et al., 2002), changes in FGF signaling might be important for the regulation of endogenous *Gdf11* expression. However, *Fgf8* expression has only been quantitatively analyzed after *Gdf11* expression onset in chick embryos (Denans et al., 2015), while how FGF signaling fluctuates prior to *Gdf11* expression has not been reported. Therefore, it is necessary to quantitatively analyze FGF signaling levels before and after *Gdf11* expression onset.

The expression level of *Gdf11* was significantly decreased at E8.5 at which endogenous *Gdf11* expression initiates in *Gdf11^ΔHCR/ΔHCR^* mice without affecting its initiation timing. Furthermore, its expression level was further decreased at E8.5 in *Gdf11^ΔHCR/-^* mice, suggesting that HCR regulates the level of *Gdf11* expression rather than the initiation timing of *Gdf11* expression. In our study, the expression level of *Gdf11* correlated with the position of the sacral-hindlimb unit. Embryos with lower levels of *Gdf11* expression showed a more posterior shift in the sacral-hindlimb unit. In *Gdf11^ΔHCR/ΔHCR^* mice, reduction of *Gdf11* expression level was continued from E8.5 to E9.5, implying that position of the sacral-hindlimb unit is determined by both level of GDF11 produced and time of exposure to GDF11 after *Gdf11* expression initiated (Figure 7).

The phenotype of *Gdf11^ΔHCR/ΔHCR^* mice fails to mimic that of *Gdf11* ^−/−^ mice (McPherron et al., 1999) (our *Gdf11* ^−/−^ mice line) (Supplementary Figure S4). This suggests the presence of *Gdf11* enhancers other than HCR. Other *Gdf11* enhancers, either individually or in conjunction with HCR, would regulate the initiation time and the level of *Gdf11* expression. Our results suggest the existence of species-specific *Gdf11* enhancers, because there is no conserved region within 30 kb of the *Gdf11* locus. Because the timing of *Gdf11* expression initiation differs according to the position of the sacral-hindlimb unit in tetrapods (Matsubara et al., 2017), interspecies differences in enhancer activity are thought to produce differences in the initiation of *Gdf11* expression and the levels of GDF11 produced, leading to diversity in the position of the sacral-hindlimb unit. Several reports have suggested that morphological differences among species are caused by changes in gene expression patterns due to differences in enhancer sequences and activity (Shashikant et al., 1998; Cretekos et al., 2008). For example, bat-accelerated regions (BARs), which evolved rapidly in the bat genome, are conserved in other vertebrate species. In BARs, the number of TFBSs required for limb bud development varies. Reporter assays have revealed that some BARs show reporter gene expression patterns different from those of mouse orthologous sequences (Booker et al., 2016). To elucidate the mechanism responsible for the diversity in the position of the sacral-hindlimb unit, it is necessary to identify predicted species-specific *Gdf11* enhancers and analyze their functions.

We also focused on WNT signaling as a candidate upstream factor that regulates *Gdf11* expression in the HCR sequence. The enhancer activity of the HCR was significantly decreased after CA-*β-catenin* overexpression, suggesting that upregulation of WNT signaling negatively regulated the enhancer activity of the HCR. However, even when WNT signaling was manipulated, there was no significant effect on the expression of endogenous *Gdf11*, suggesting that WNT signaling does not regulate endogenous *Gdf11* expression. Furthermore, CA-*β-catenin* overexpression did not induce downregulation of endogenous *Gdf11* expression (Supplementary Figure S5). These results suggest that WNT signaling negatively regulates HCR enhancer activity; however, it does not affect endogenous *Gdf11* expression. We also investigated whether the onset of *Gdf11* expression and the level of endogenous *Gdf11* expression after its induction are altered by the treatment combination of FGF and WNT signaling agonists/inhibitors. However, these treatments did not affect *Gdf11* expression (Supplementary Figure S6).

There were many predicted TFBSs downstream of FGF and WNT signaling in the HCR (Figure 5A); however, these signaling pathways did not primarily control the enhancer activity of HCR. This suggests the existence of other transcription factors that control the HCR enhancer activity. The discovery of other transcription factors that positively regulate HCR enhancer activity and other enhancers in addition to HCR will help clarify the mechanism underlying the positioning of the sacral hindlimb unit along the anterior-posterior axis through *Gdf11* expression.

## Data Availability

The datasets presented in this study can be found in Mendeley Data online repository. https://data.mendeley.com/datasets/ddnkcgm7h4/1, doi: 10.17632/ddnkcgm7h4.1 Key resources used in this study is listed in Table 3. Key resources used in this study.
