# Supplementary figures and images for: Functional analysis of a first hindlimb positioning enhancer via Gdf11 expression
Source: Front Cell Dev Biol. 2024 Mar 15;12:1302141. doi: 10.3389/fcell.2024.1302141 (PMC10978735; doi:10.3389/fcell.2024.1302141)

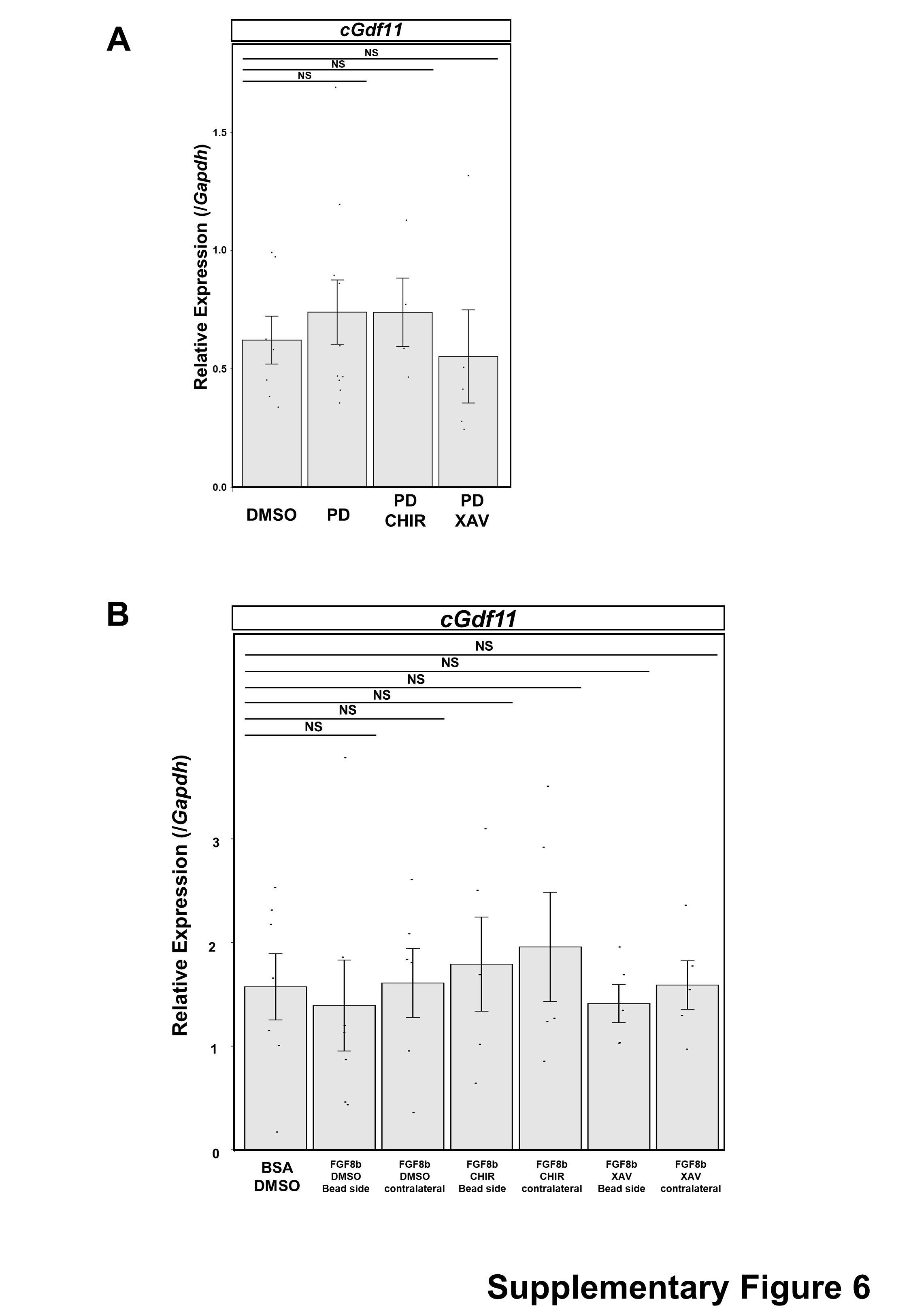

Supplement: Supplementary file 1 [file Image6.TIF]

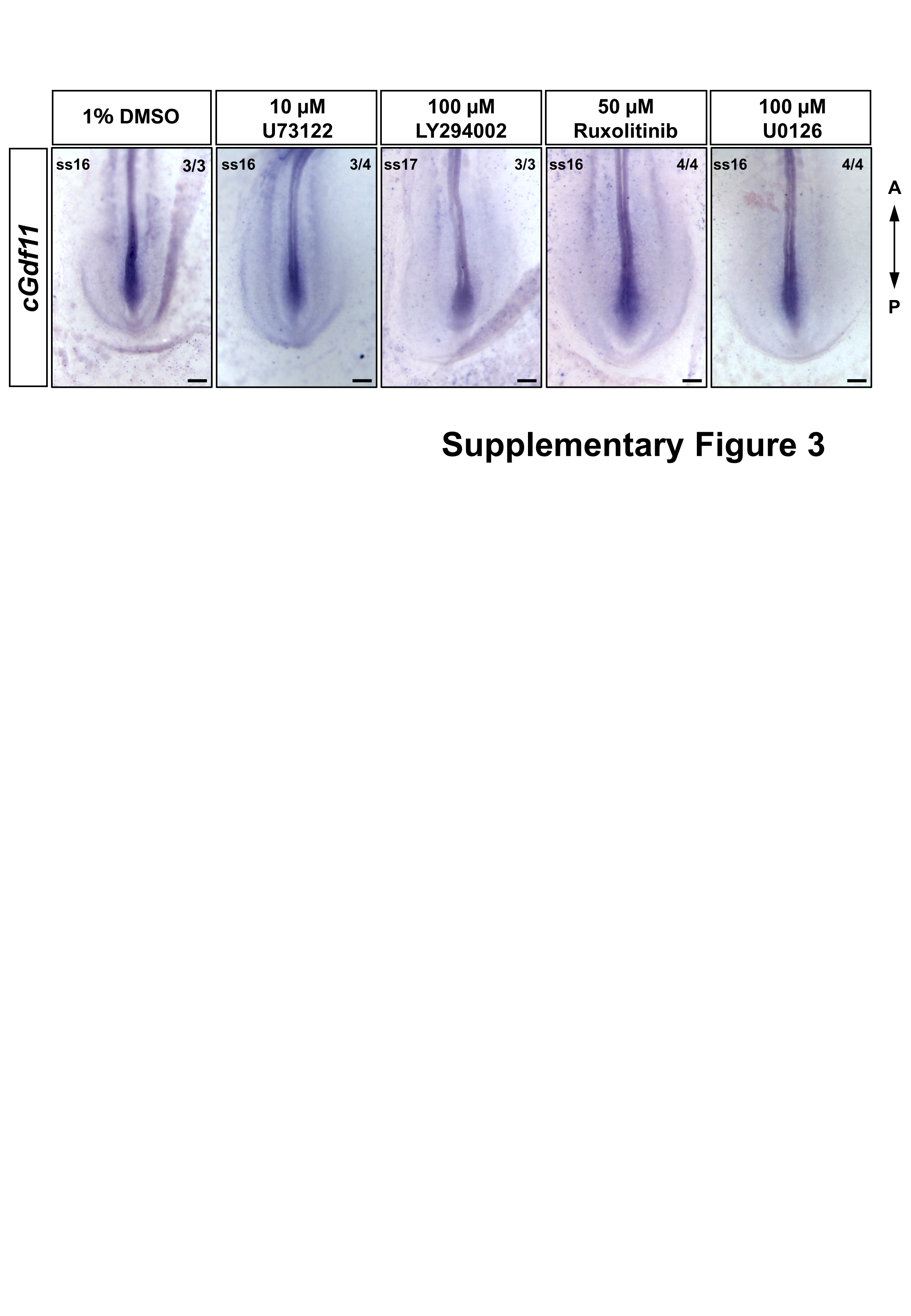

Supplement: Supplementary file 2 [file Image3.TIF]

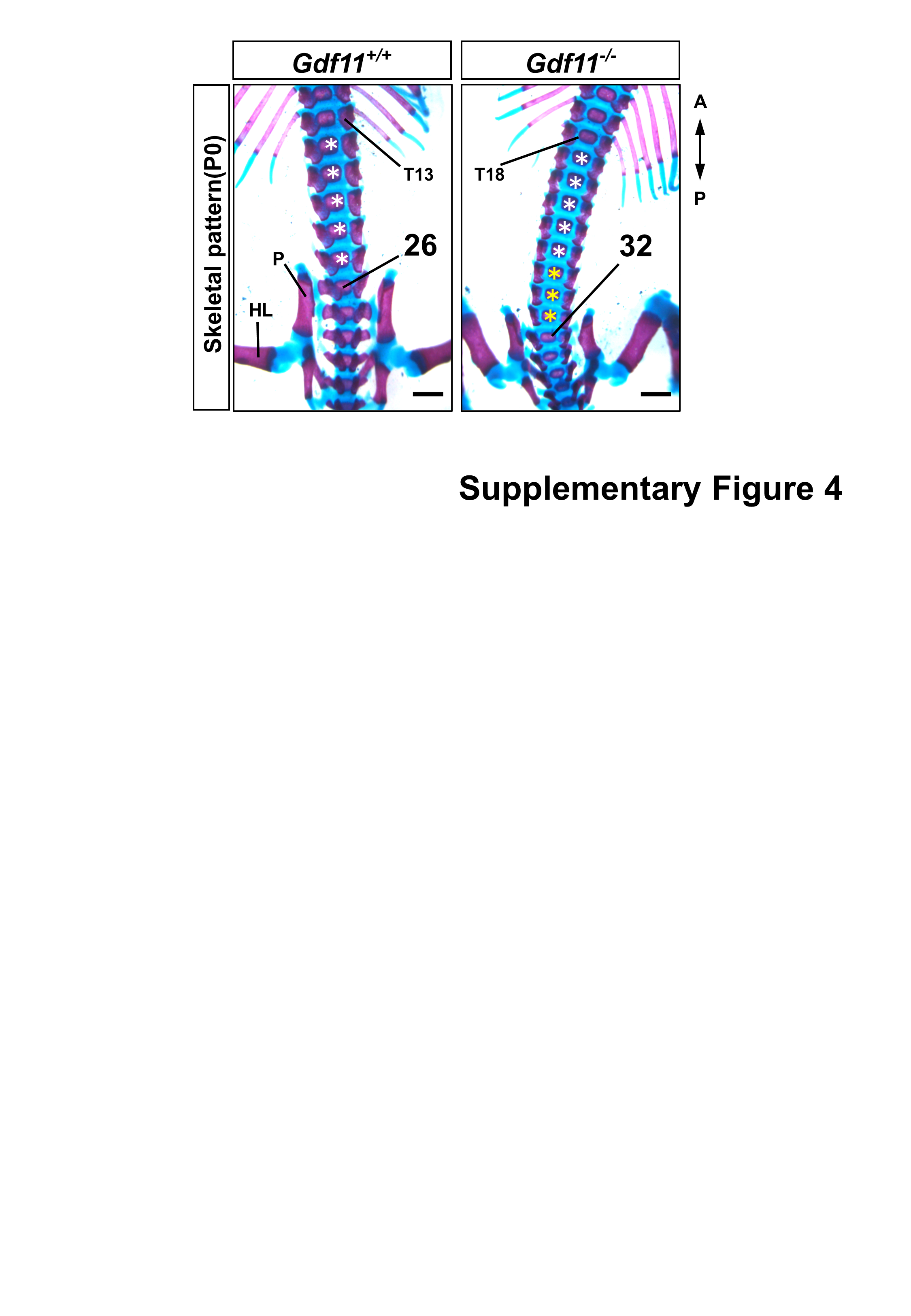

Supplement: Supplementary file 3 [file Image4.TIF]

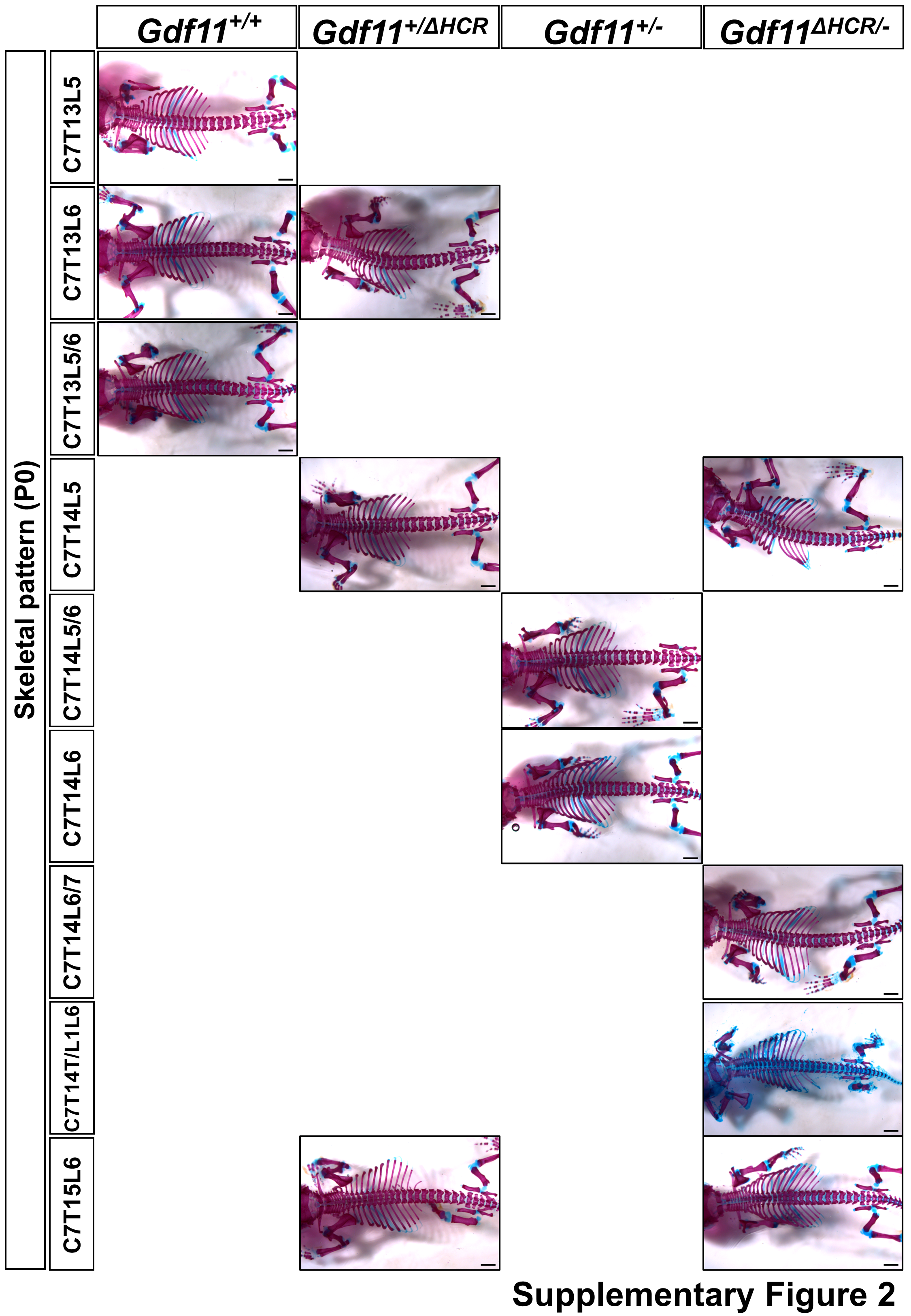

Supplement: Supplementary file 4 [file Image2.TIF]

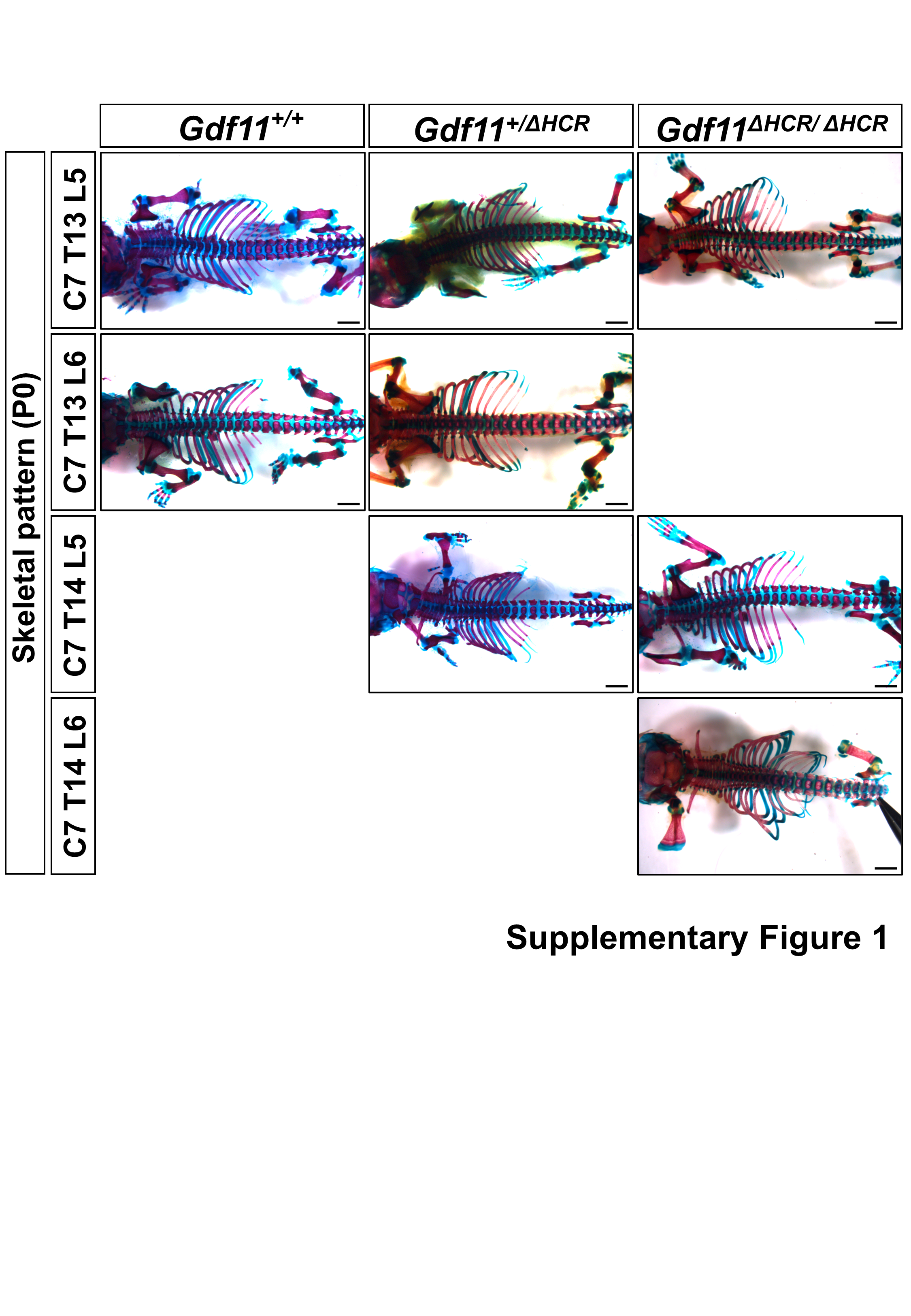

Supplement: Supplementary file 5 [file Image1.TIF]

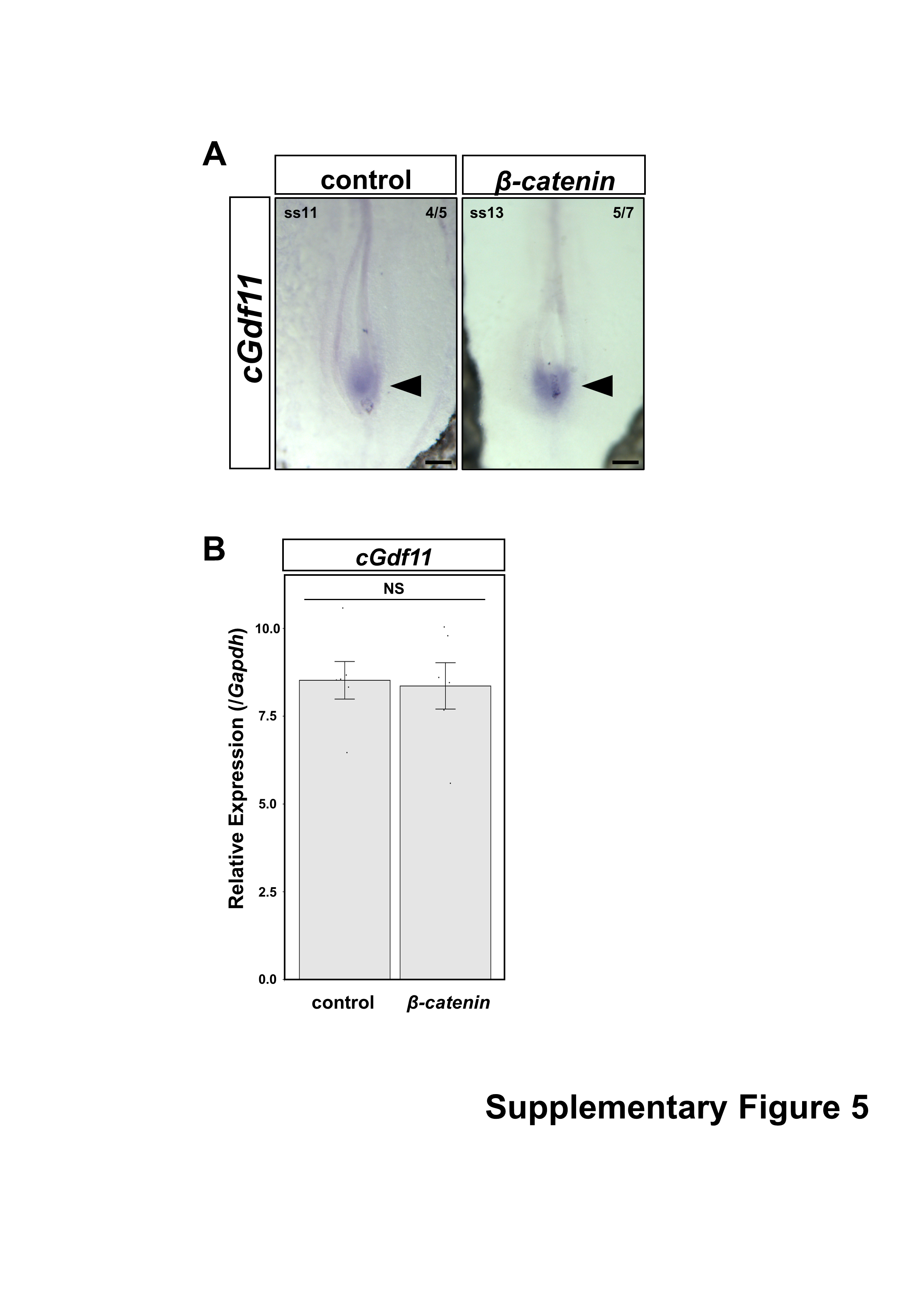

Supplement: Supplementary file 6 [file Image5.TIF]
